# Supplementary material for: Force-dependent allostery of the α-catenin actin-binding domain controls adherens junction dynamics and functions
Source: Nat Commun. 2018 Nov 30;9:5121. doi: 10.1038/s41467-018-07481-7 (PMC6269467; doi:10.1038/s41467-018-07481-7)
Supplement: Supplementary file 1 — Supplementary Information [file 41467_2018_7481_MOESM1_ESM.pdf]

Force-dependent allostery of the α-catenin actin-binding domain controls adherens junction dynamics and functions

Ishiyama et al.

Supplementary Information

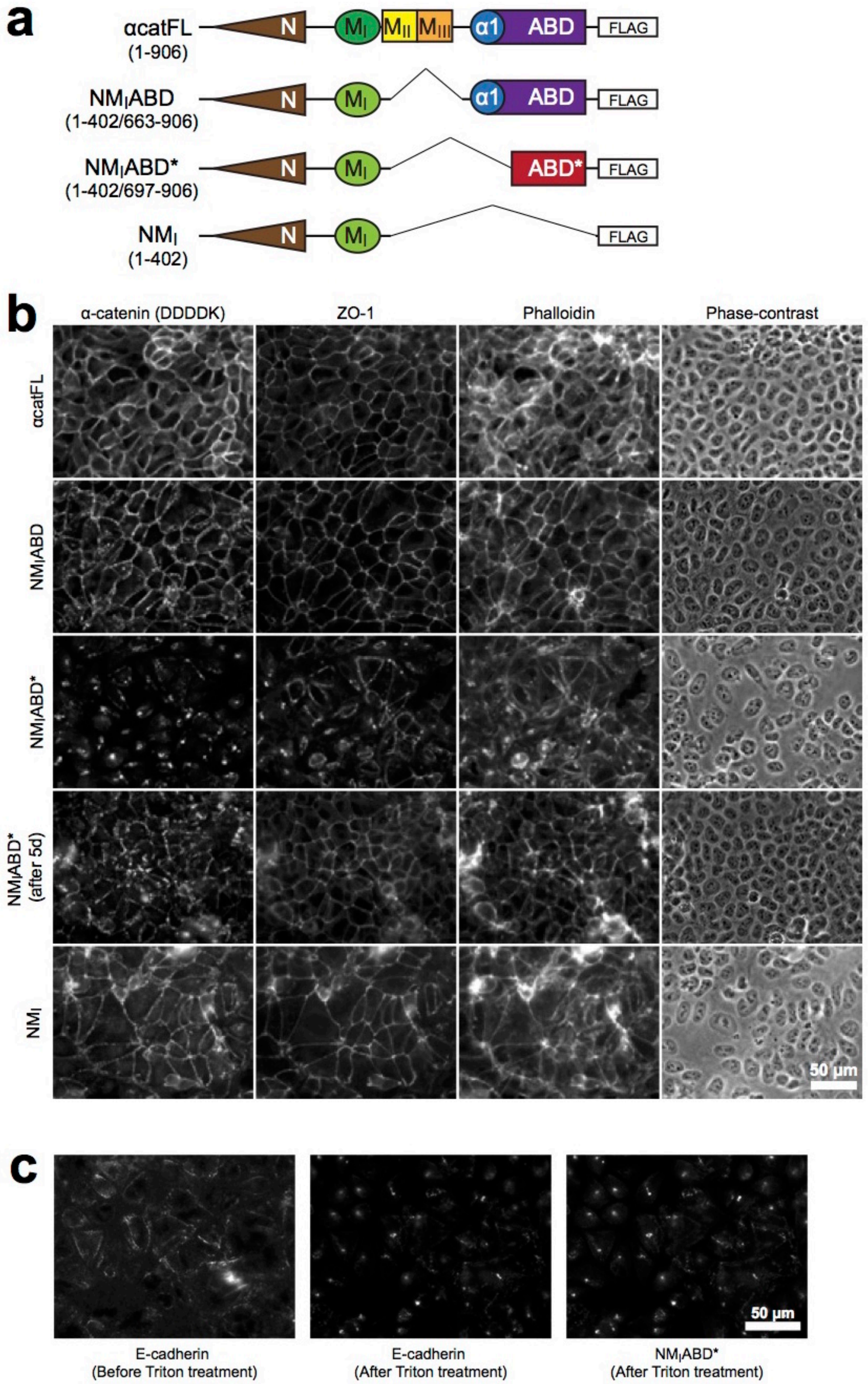

Supplementary Figure 1: Cadherin-catenin complexes trapped intracellularly with F-actin during cell-cell junction remodelling**.**

a. Schematic diagrams of αE-catenin variants. αcatFL: full-length αE-catenin (residues 1-906). N: N-terminal dimerization domain (residues 1-262). M_I: M_I region (residues 290-395) contains

a cryptic vinculin-binding site (VBS; residues 305-352) that becomes constitutively active when the autoinhibitory M_{II-III} regions are absent. ABD: actin-binding domain (residues 663-906). α1: the α1-helix (residues 669-675) of ABD. ABD*: an N-terminally truncated ABD (residues 697-906), which is missing α1-helix and a part of α2-helix. All α-catenin constructs contain a C-terminal FLAG tag.

b. R2/7 cells stably expressing α-catenin variants were trypsinized, replated at 30% confluency and imaged after 2d (additional images were taken after 5d for NM_IABD*). Since full-length α-catenin can interact with F-actin both directly and indirectly (e.g., via vinculin) in a force-dependent manner, we selectively minimized the force-dependency of indirect F-actin binding by deleting the autoinhibitory M_{II-III} regions of α-catenin to constitutively activate the M_I/VBS region. Cells expressing various α-catenin mutants (αcatFL, NM_IABD, NM_IABD* and NM_I) were compared to examine the effects of ABD deletions on the direct α-catenin-F-actin interaction during cell-cell junction remodelling. Whereas cells expressing NM_IABD or NM_I formed AJs at a similar pace as cells expressing αcatFL (2 d), cells expressing NM_IABD* showed cadherin-catenin complexes trapped intracellularly with F-actin (2d) and required additional time to establish tight junction network stained with ZO-1 antibody (5d).

c. Cadherin-catenin clusters found in R2/7 cells expressing NM_IABD* (as shown in b) distribute intracellularly 2 days after replating. Before triton treatment, ECCD2 that recognizes the extracellular domain of E-cadherin was applied and it stained E-cadherin expressed only on cell surface (left). After triton treatment, clone36/E-cadherin that recognizes the cytoplasmic domain of E-cadherin stained clusters in the cytoplasm (middle), suggesting that these structures are likely isolated in the cytoplasm. NM_IABD* colocalizes with the E-cadherin cluster (right).

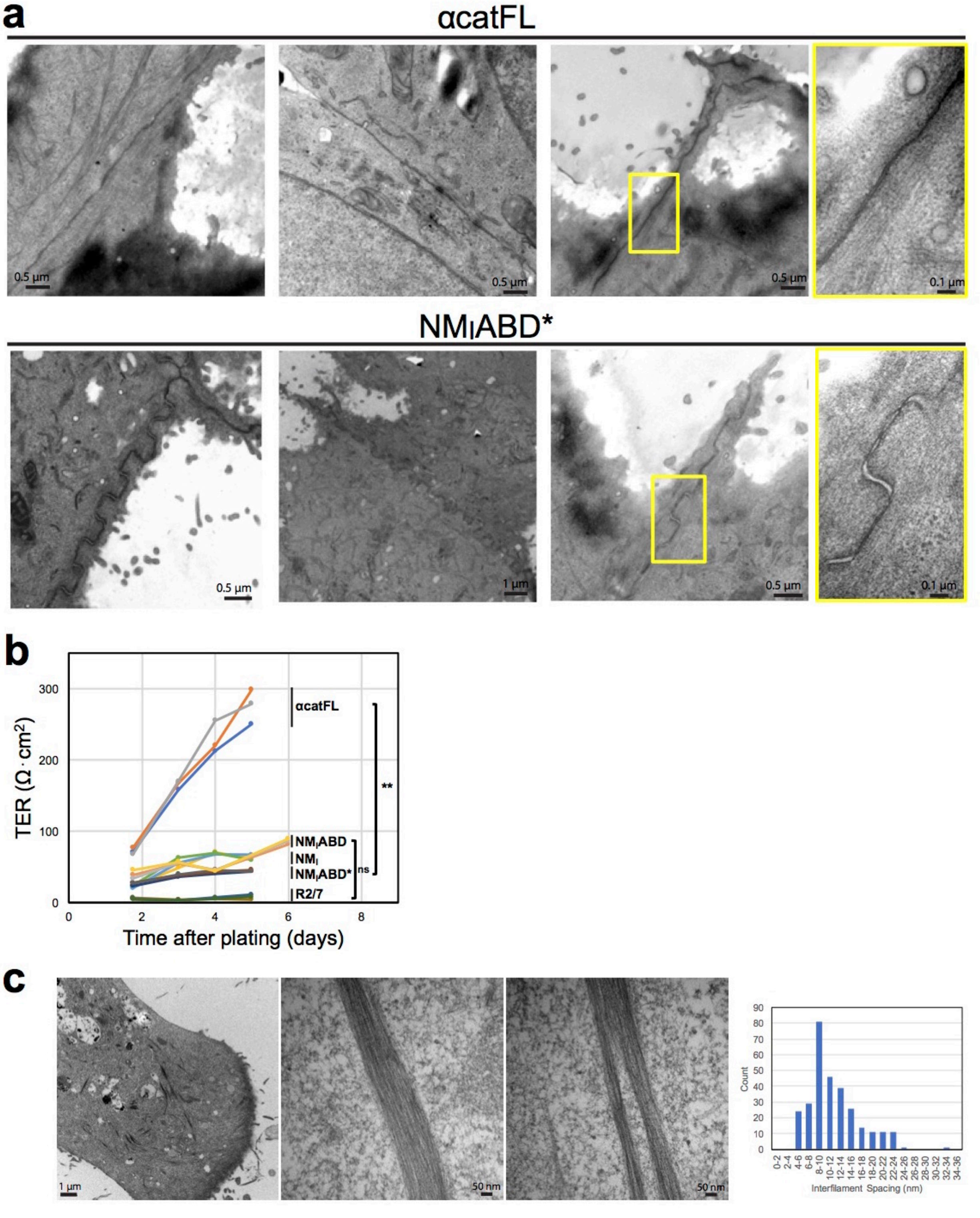

Supplementary Figure 2: An N-terminal truncation of the α-catenin ABD contributes to square-wave-like intercellular junctions and the actin rod formation.

a. Electron micrographs of cell-cell junctions formed by R2/7 cells expressing αcatFL or NM_IABD^*. While αcatFL-expressing cells formed continuous linear cell junctions, NM_IABD^*-expressing cells formed intercellular junctions in a square-wave pattern.

b. Transepithelial electric resistance (TER) measurements of R2/7 cells expressing αE-catenin variants over 5 days. Compared with αEcatFL, cells expressing other αE-catenin variants displayed lower TER, indicating aberrant tight junction formation and/or function. Significance by ANOVA. N=3.
c. Electron micrographs of actin rods formed in R2/7 cells expressing ABD* (left). These actin rods consist of tightly packed F-actin bundles (middle) with the average inter-filament spacing of 7-8 nm (right).

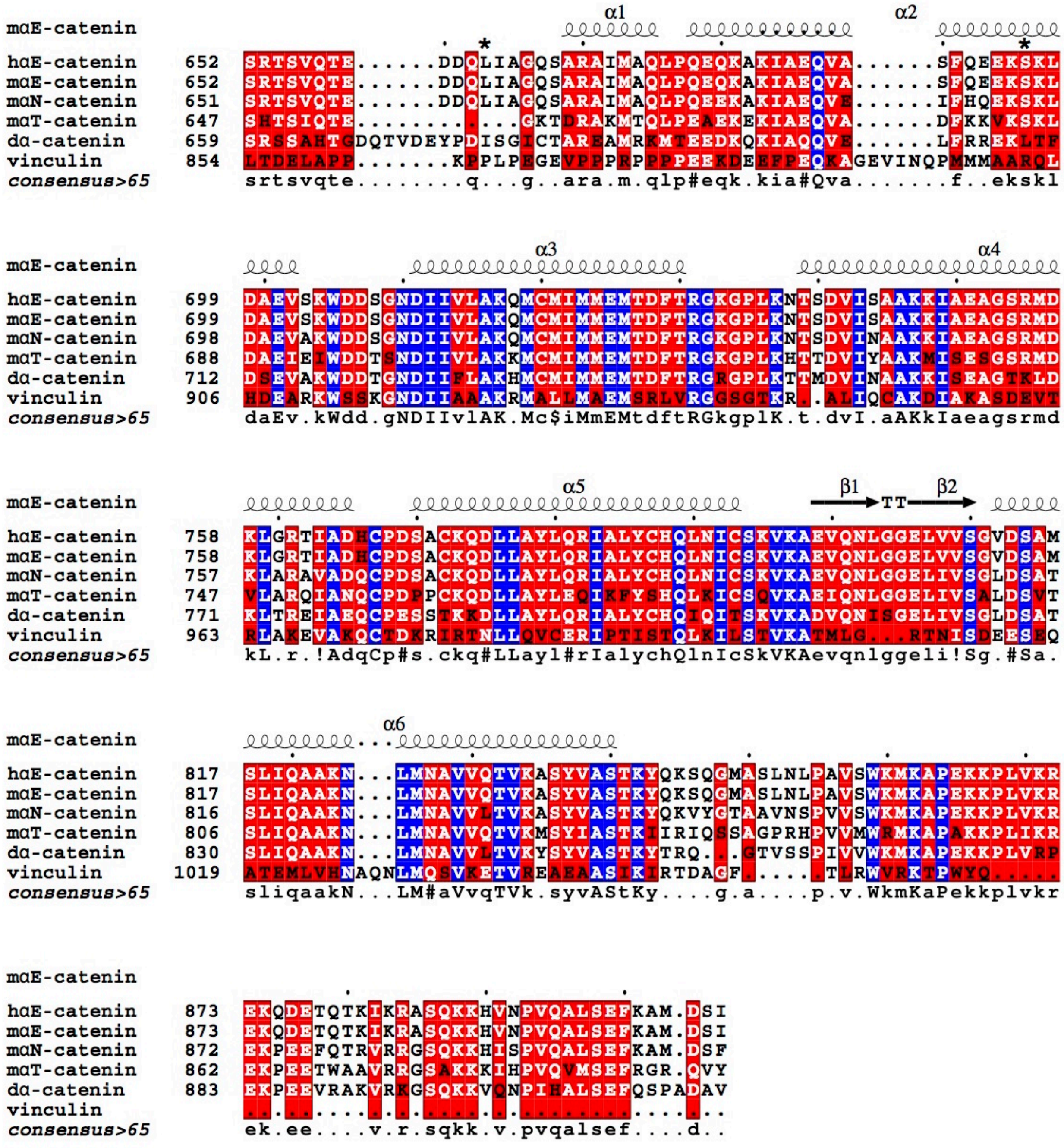

Supplementary Figure 3: Multiple sequence alignment of the α-catenin/vinculin ABDs. Multiple sequence alignment of human αE-catenin, mouse αE-catenin, mouse αN-catenin, mouse αT-catenin, *Drosophila* α-catenin, and human vinculin. The secondary structure elements shown are from the crystal structure of αNcat-ABD-WT (PDB ID: 4K1O). Asterisks (*) mark the αE-catenin residues 663 and 696. Blue box: strictly conserved residues. Red box: highly conserved residues.

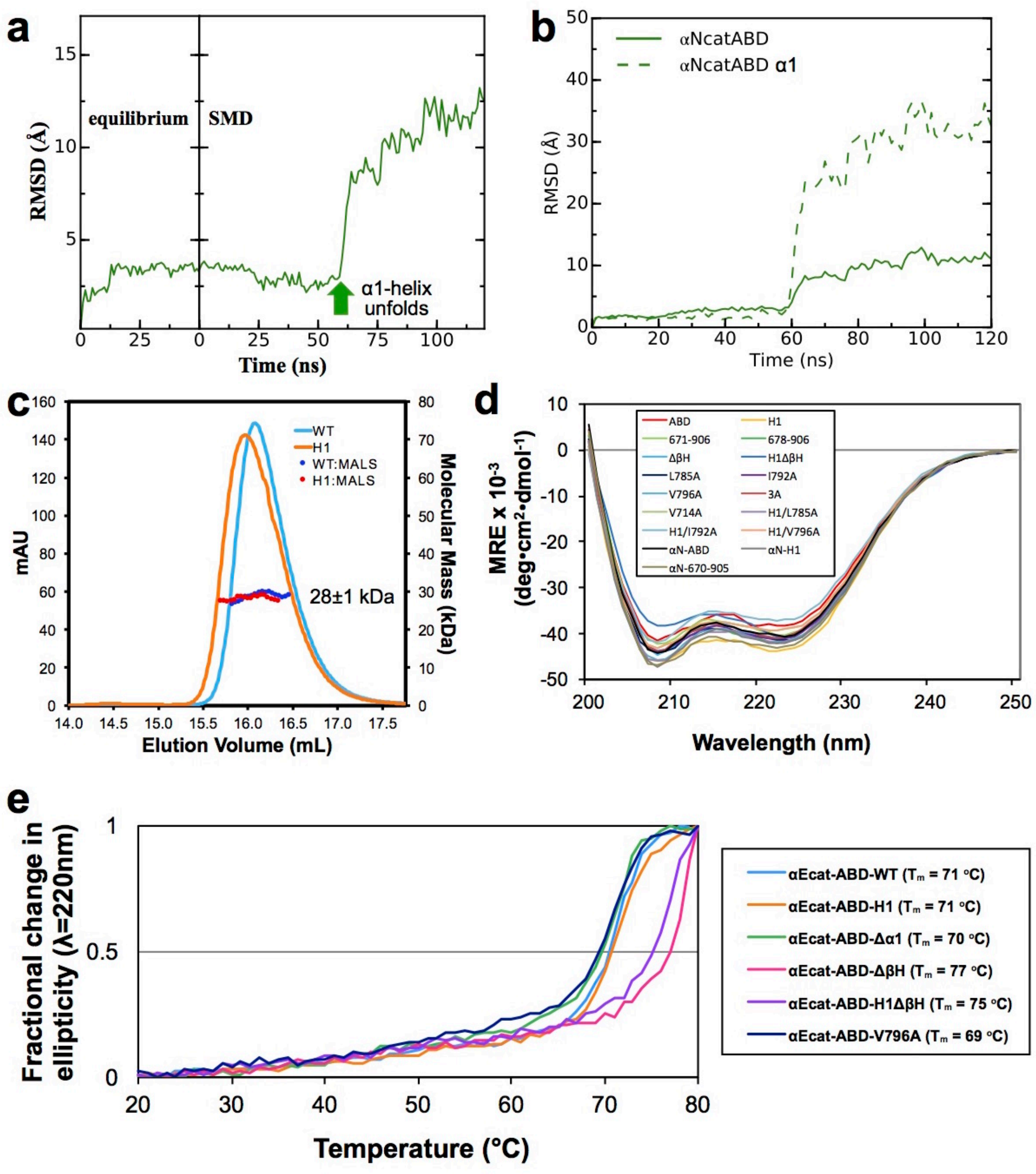

Supplementary Figure 4: Structural stability of the α-catenin ABD.

a. Root-mean-square deviation (RMSD) from the crystal structure of the αNcat-ABD-WT during 50-ns equilibrium molecular dynamics (MD) and subsequent 120-ns constant-force SMD simulations. A sudden increase in the RMSD value coincides with the unfolding of α1-helix at 60 ns (green arrow).

b. Plots of RMSD from the equilibrated ABD structure (the initial state of the constant-force SMD simulation in **a**) for the entire ABD (green solid line) and the α1-helix (green dashed line) during the constant-force SMD simulation of αNcat-ABD-WT.

c. SEC-MALS profiles of αEcat-ABD-WT and αEcat-ABD-H1 obtained with the 5 mg/mL protein samples. The MALS-based estimation of the molecular masses of WT and H1 (28±1 kDa) are consistent with the theoretical mass of the αEcat-ABD-WT monomer (28,522 *Da*).
d. CD spectra of αEcat- and αNcat-ABD variants. Samples are αEcat-ABDs, except for three αNcat-ABDs denoted by "αN-" label.
e. Thermal unfolding plots of αEcat-ABD variants monitored by far-UV CD changes at 220 nm. The melting temperature (Tm) of αEcat-ABD was not affected by various mutations, including H1, ΔβH and V796A.

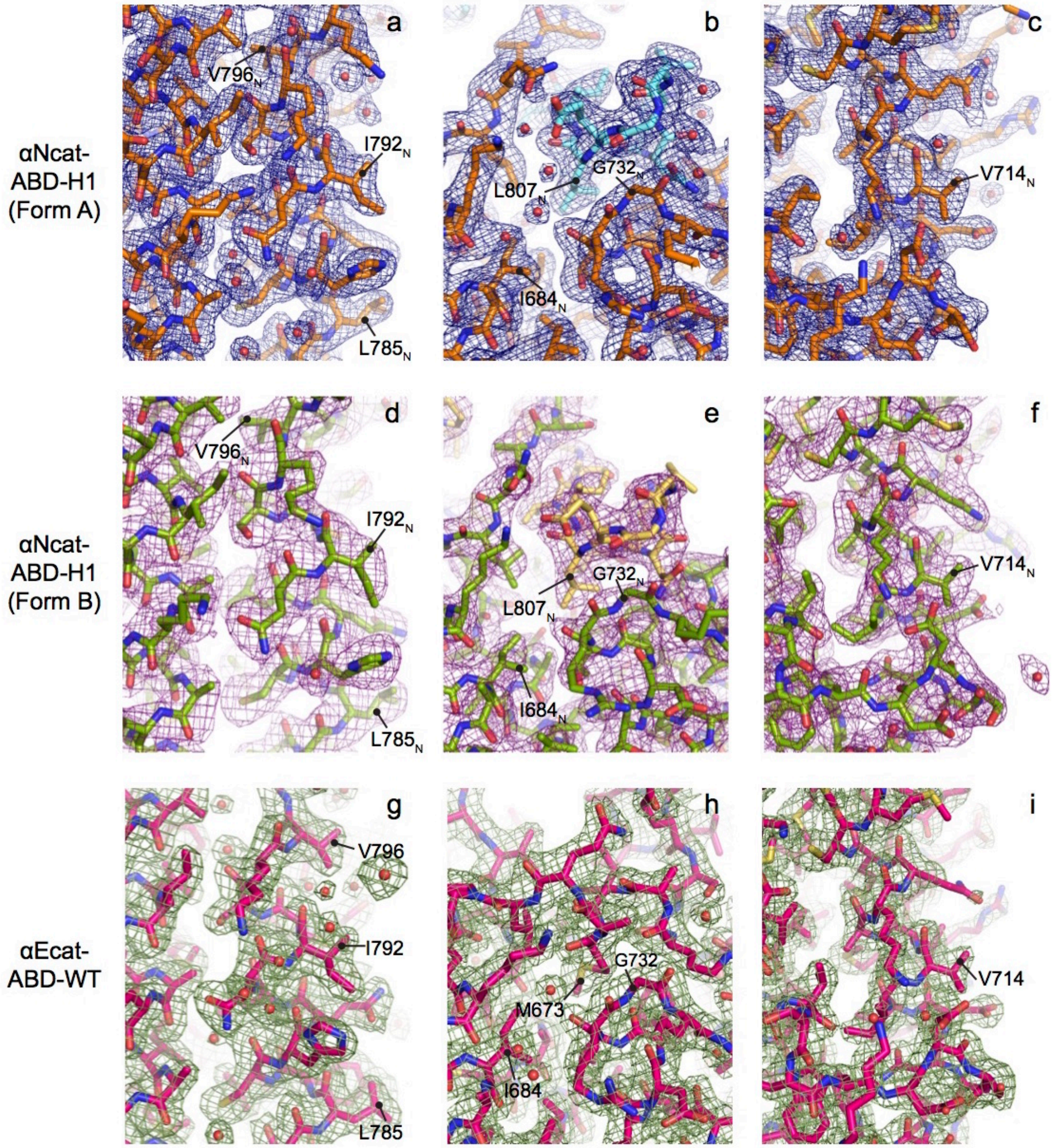

Supplementary Figure 5: Electron density maps of αcat-ABD crystal structures. **Top:** The 2mFo-DFc map (1σ) of the αNcat-ABD-H1 form A crystal structure around the actin-binding site (a), βH-dimer interface (b) and V714 (c). **Middle:** The 2mFo-DFc map (1σ) of the αNcat-ABD-H1 form B crystal structure in similar views as top panels are shown as d, e and f. **Bottom:** The 2mFo-DFc map (1σ) of the αEcat-ABD-WT crystal structure in similar views as top panels are shown as g, h and i.

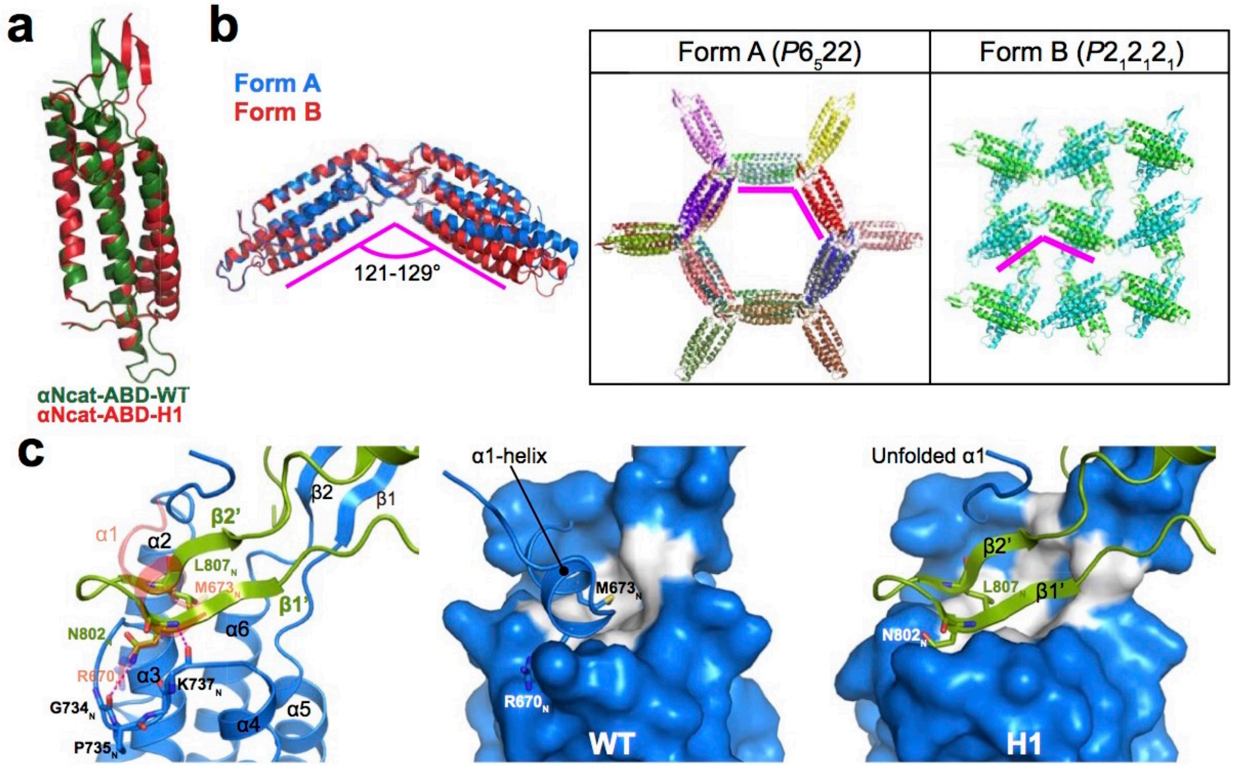

Supplementary Figure 6: Structural studies of the αcat-ABD dimer interface.

a. Superposition of αNcat-ABD-WT and αNcat-ABD-H1 crystal structures. All crystallographically unique structures of αNcat-ABD-H1 from two different crystal forms closely resemble the αNcat-ABD-WT structure (PDB ID: 4K1O): the RMSD of Cα atoms of 148 out 186 residues is <0.36 Å, except for the αN-catenin residues 669_N-675_N adopting an extended conformation instead of an α-helix due to the H1 mutation and the βH motif (residues 799_N-810_N).

b. Overlay of αNcat-ABD-H1 homodimer structures from crystal forms A and B. The asymmetrical unit of form A contains one molecule, thus the homodimer consists of two crystallographically related molecules, whereas in form B two αNcat-ABD-H1 molecules found in the asymmetric unit constitute the homodimer. Symmetry molecules of αNcat-ABD-H1 in two different crystal forms are shown (right). The intermolecular angle of the ABD dimer ranges from 121 to 129°.

c. The ABD dimer interface involves a hydrophobic patch exposed by α1-helix unfolding. The left panel shows a slightly rotated view of Fig. 2b. A surface rendition of the αNcat-ABD-WT residues 677_N-906_N with the α1-helix in cartoon (middle) helps to identify a hydrophobic patch (white) beneath the α1-helix. At the dimer interface, the βH of each protomer interacts with the hydrophobic patch on the opposite protomer in a reciprocal manner (right). Interestingly, the βH residue $L807_N$ mimics a key hydrophobic interaction between $M673_N$ of α1-helix and the hydrophobic patch in the αNcat-ABD-WT structure (left).

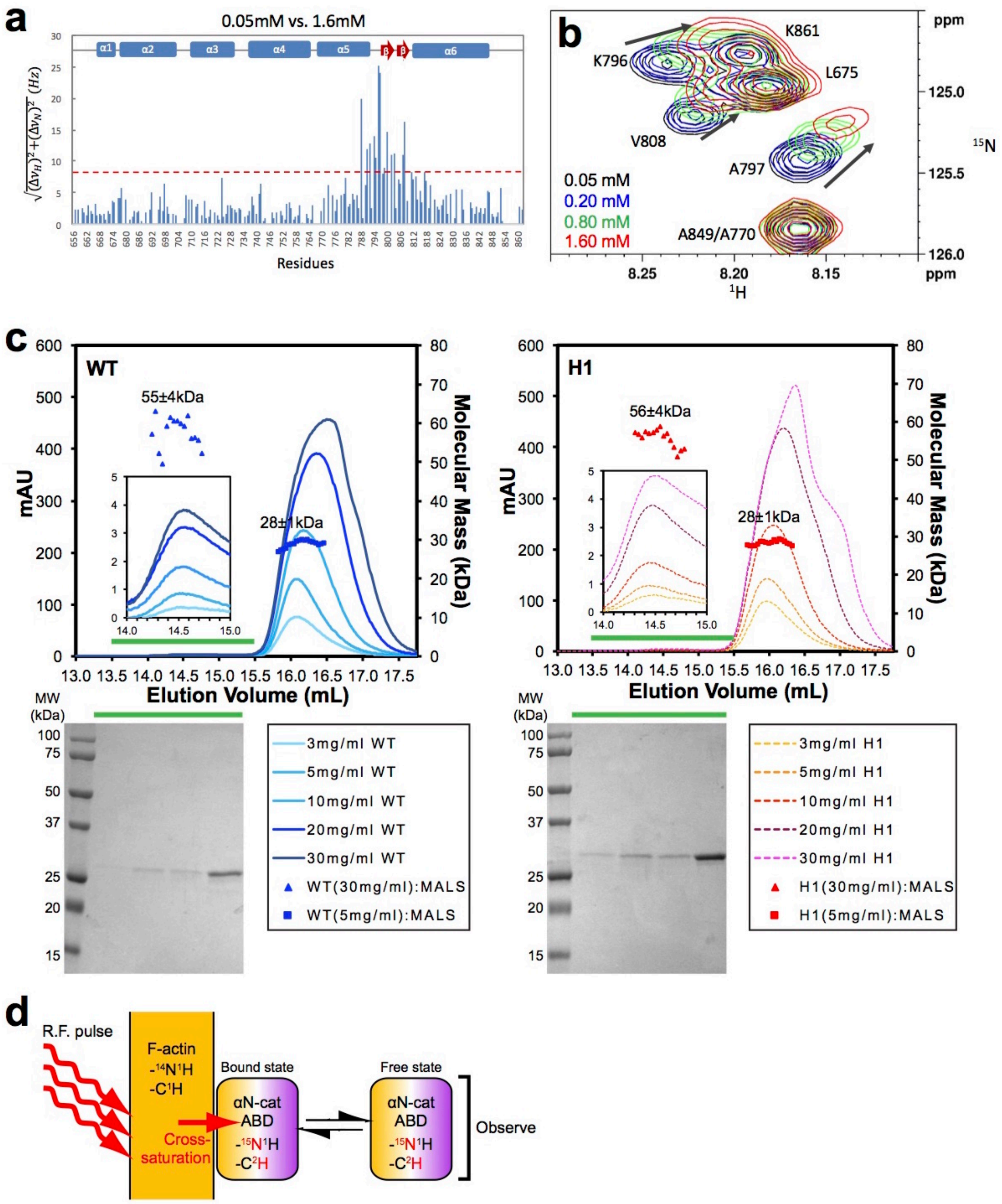

Supplementary Figure 7: Weak homodimerization of αcat-ABD.

a. Plots of chemical shift perturbations (CSPs) of αNcat-ABD-H1 at two different sample concentrations (0.05 mM and 1.6 mM) show that concentration-dependent perturbations mostly occurred in the βH motif.

b. An overlay of ^{1}H-^{15}N HSQC spectra of ^{15}N-αNcat-ABD-H1 collected at different titration steps from 0.05 mM to 1.60 mM.
c. SEC-MALS profiles of αEcat-ABD. The elution profiles of αEcat-ABD-WT (left) and αEcat-ABD-H1 (right) at different concentrations (3 mg/ml to 30 mg/ml) are plotted with the MALS analysis results showing the estimated molecular mass of αcat-ABD monomers and dimers. Significance by ANOVA. N = 3. The dimer peak fractions (green line) collected from the 30 gm/mL SEC runs were analyzed by SDS-PAGE.
d**.** A scheme of TCS experiments performed with unlabeled F-actin and $^{15}N/^{2}H$-labeled αNcat-ABD-WT.

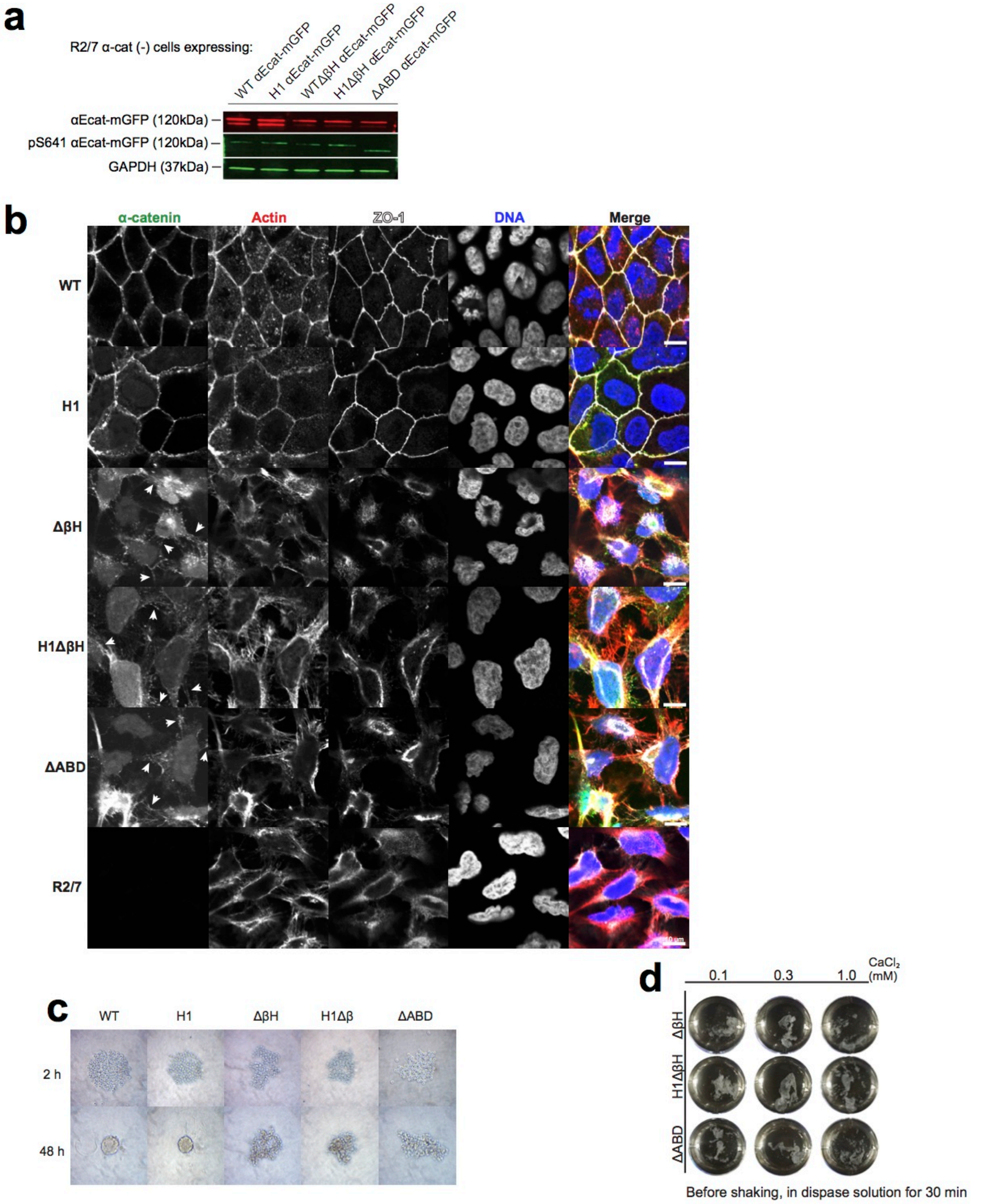

Supplementary Figure 8: αcat-ABD is critical for the formation of multicellular structures.
a. Immunoblots of R2/7 cells expressing α-catenin variants tagged with mGFP. pS641 antibody is specific for αE-catenin containing phosphorylated Ser641.
b. Confocal images of R2/7 cells expressing α-catenin variants tagged with mGFP. Actin filaments are stained with Phalloidin. ZO-1 is visualized by immunostaining. DNA is stained

with Hoechst 33342. Colocalization of α-catenin and F-actin at AJs was readily observed in R2/7 cells expressing αEcat-WT or αEcat-H1, but not with cells expressing the ΔβH, H1ΔβH or ΔABD variant. Accumulation of α-catenin in protrusions of cells expressing the ΔβH, H1ΔβH or ΔABD variant is indicated by arrows.

c. 3D cell culture of R2/7 cells expressing α-catenin variants. Incubation of R2/7 cells (~200 cells) expressing αEcat-WT or αEcat-H1 resulted in the formation of single spheroid structure after 48 h. In comparison, R2/7 cells expressing αEcat-ΔβH, αEcat-H1ΔβH or αEcat-ΔABD mostly remained as aggregated mass of cells.

d. Dispase assay of R2/7 cells expressing α-catenin variants. Cells expressing αEcat-ΔβH, αEcat-H1ΔβH or αEcat-ΔABD resulted in extensive fragmentation of cell monolayers in dispase solution prior to mechanical disruption.

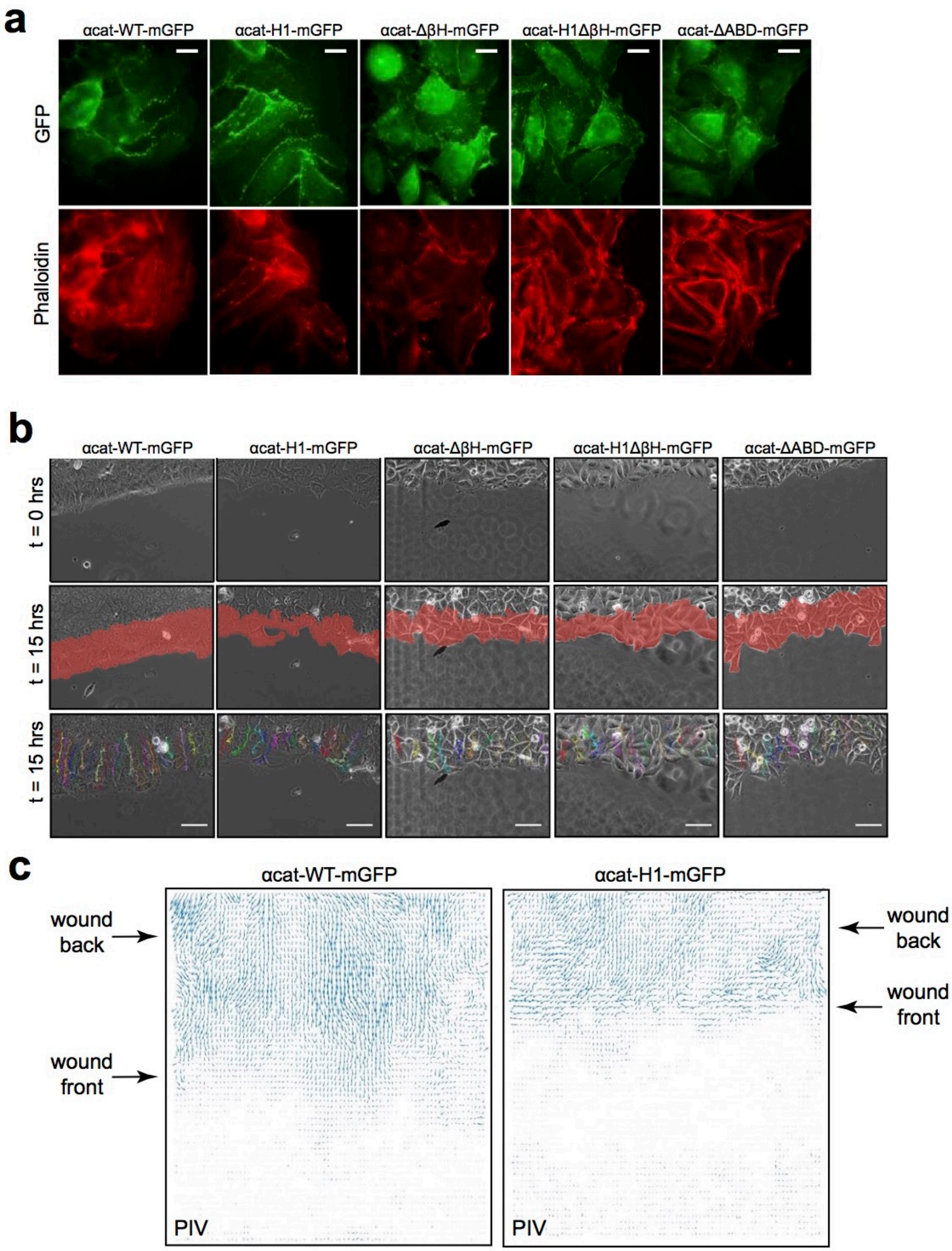

Supplementary Figure 9: Comparison of R2/7 cells expressing αEcat variants in wound healing.
a. Confocal images of R2/7 cells expressing αEcat variants at the wound fronts.

b. Scratch wound healing assay with R2/7 cells expressing αEcat variants. Cells in monolayers lacking cell-cell adhesion move in a less coordinated fashion, whereas the αEcat-H1 wound front simply moves more slowly (see also Supplementary Movie 4).
c. Particle image velocimetry (PIV) analysis averaged from 3 αEcat-WT (left) or 4 αEcat-H1 (right) scratch wounds from two independent biological replicates. Note that as with the manual tracking method of wound-proximal cells in Fig. 3d and Supplementary Fig. 9b, this automated method shows that vector direction, as opposed to size (*i.e.,* cell speed)- particularly at the wound front- is most different between αEcat-WT versus αEcat-H1-expressing cells. For visualization, vector arrow scale is 2x, which corresponds to 1-2 microns per 10 minute interval.

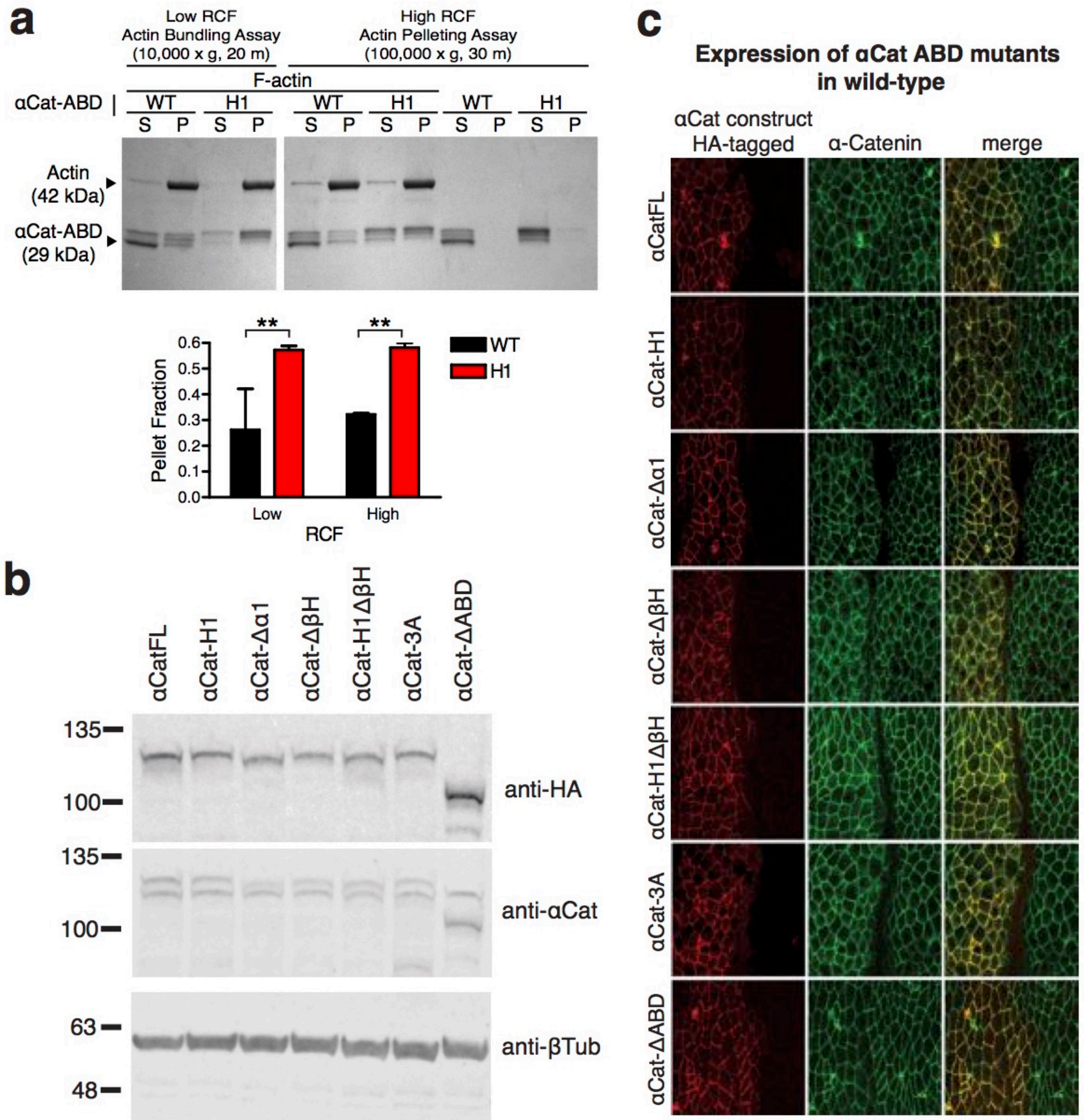

Supplementary Figure 10: Characterization of *Drosophila* α-Cat ABD mutants.

a. *In vitro* actin-binding (high RCF) and actin-bundling (low RCF) assay results of *Drosophila* αCat-ABD-WT and αCat-ABD-H1 proteins. Data are presented as mean ± SEM (N = 3). Significance by ANOVA; $^{**}P < 0.01$.

b. Immunoblot analysis of HA-tagged αCatFL and α-Cat ABD mutants expressed in *Drosophila* embryos with *da-Gal4*. Proteins are detected with antibodies directed against HA, αCat, and α-Tubulin as a loading control. Note that transgenic proteins show similar levels to endogenous α-Cat.

c. Expression of HA-tagged αCatFL and αCat ABD mutants in the epidermis of wild-type embryos at stage 15. Expression is driven in stripes by *paired-Gal4*. Embryos were stained for HA (red) and α-Cat (green). All αCat variants are enriched at AJs.

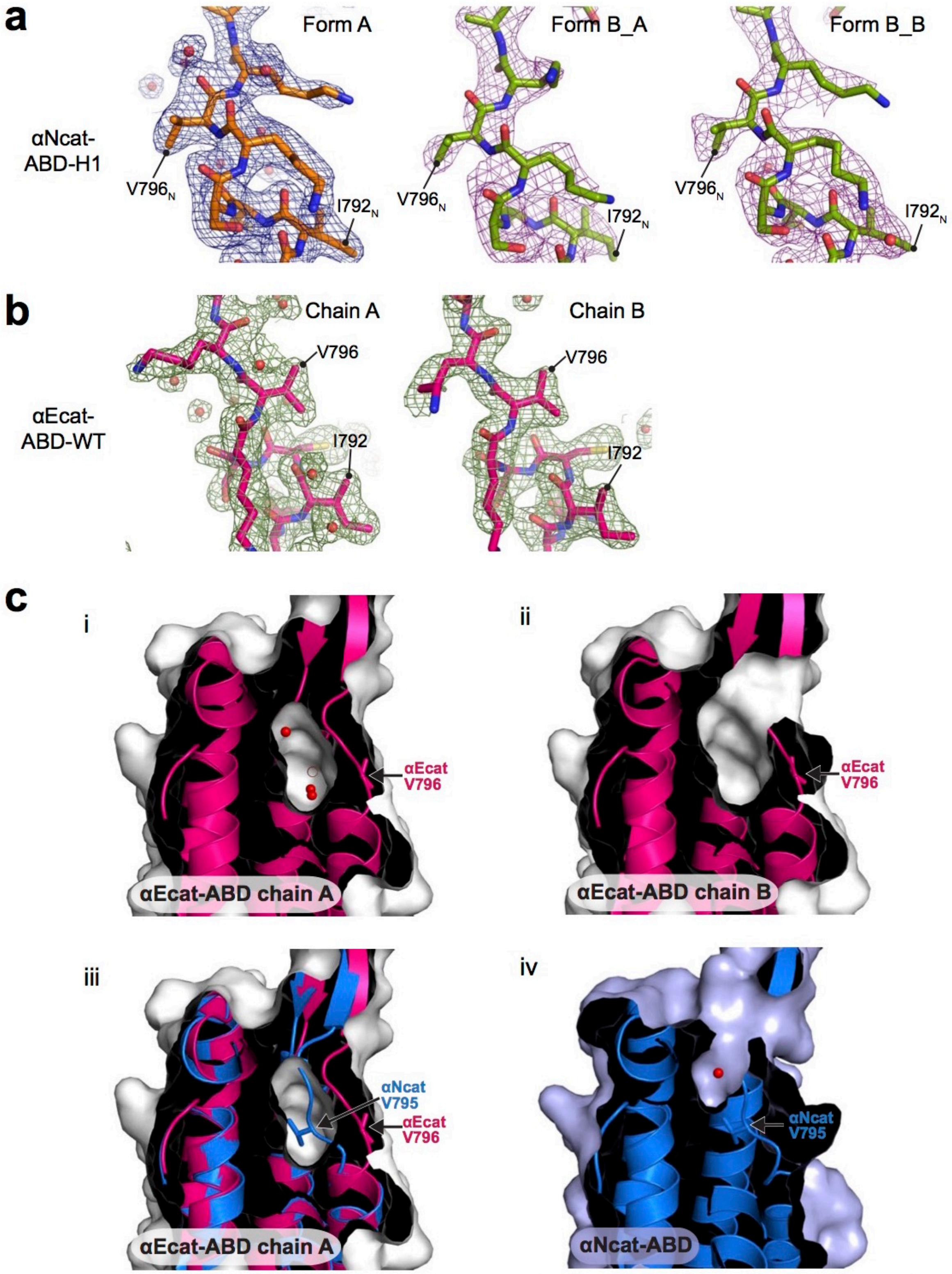

Supplementary Figure 11: Cryptic and exposed states of the actin binding site residue V796.
a. Simulated annealing-omit maps (contoured at 1σ; mesh) around $V796_N$ of αNcat-ABD-H1 show the cryptic conformational state of this residue in all three crystallographically independent structures from forms A and B.

b. The initial electron density map obtained from bromide-SAD phasing around V796 of αEcat-ABD-WT (contoured at 1σ; mesh). It shows the exposed state of V796, which is not involved in crystal packing, in both chains of the asymmetric unit. The side chain of another hydrophobic residue, I792, is also exposed on the actin-binding surface of α5-helix.

c. Surface representation of αEcat-ABD-WT and αNcat-ABD-H1. Both αEcat-ABD-WT chains A and B contain a cavity near V796: the cavity in chain A (panel i and Fig. 5b) is closed off and contains four water molecules, whereas the cavity in chain B (panel ii) is connected to the outer surface and does not contain any solvents or symmetry-related molecules. A close resemblance between the surface representations of αEcat-ABD-WT (panel iii) and αNcat-ABD-WT (panel iv) suggests that the appearance of the cavity in αEcat-ABD-WT involves a localized conformational change of the α5-β1 linkage containing V796.

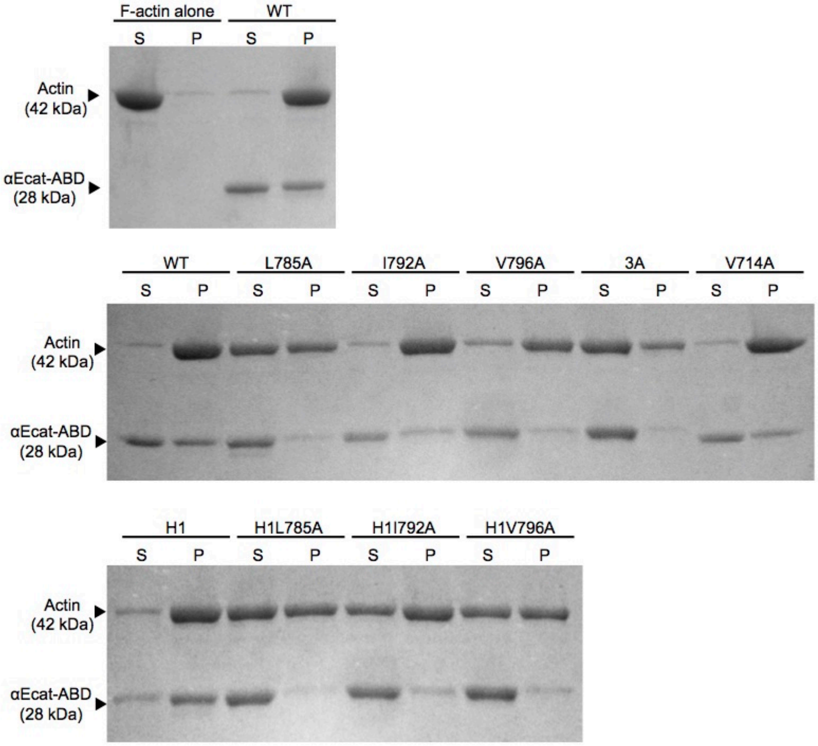

Supplementary Figure 12: *In vitro* actin bundling assays of αEcat-ABD variants.
Actin bundling was analyzed by sedimentation of the αEcat-ABD/F-actin mixture at low RCF (10,000 x g). While F-actin alone predominantly remains in the supernant (S) fraction, the incubation of F-actin with αEcat-ABD-WT or other variants resulted in the F-actin-αEcat-ABD complex cosedimented in the pellet (P) fraction.

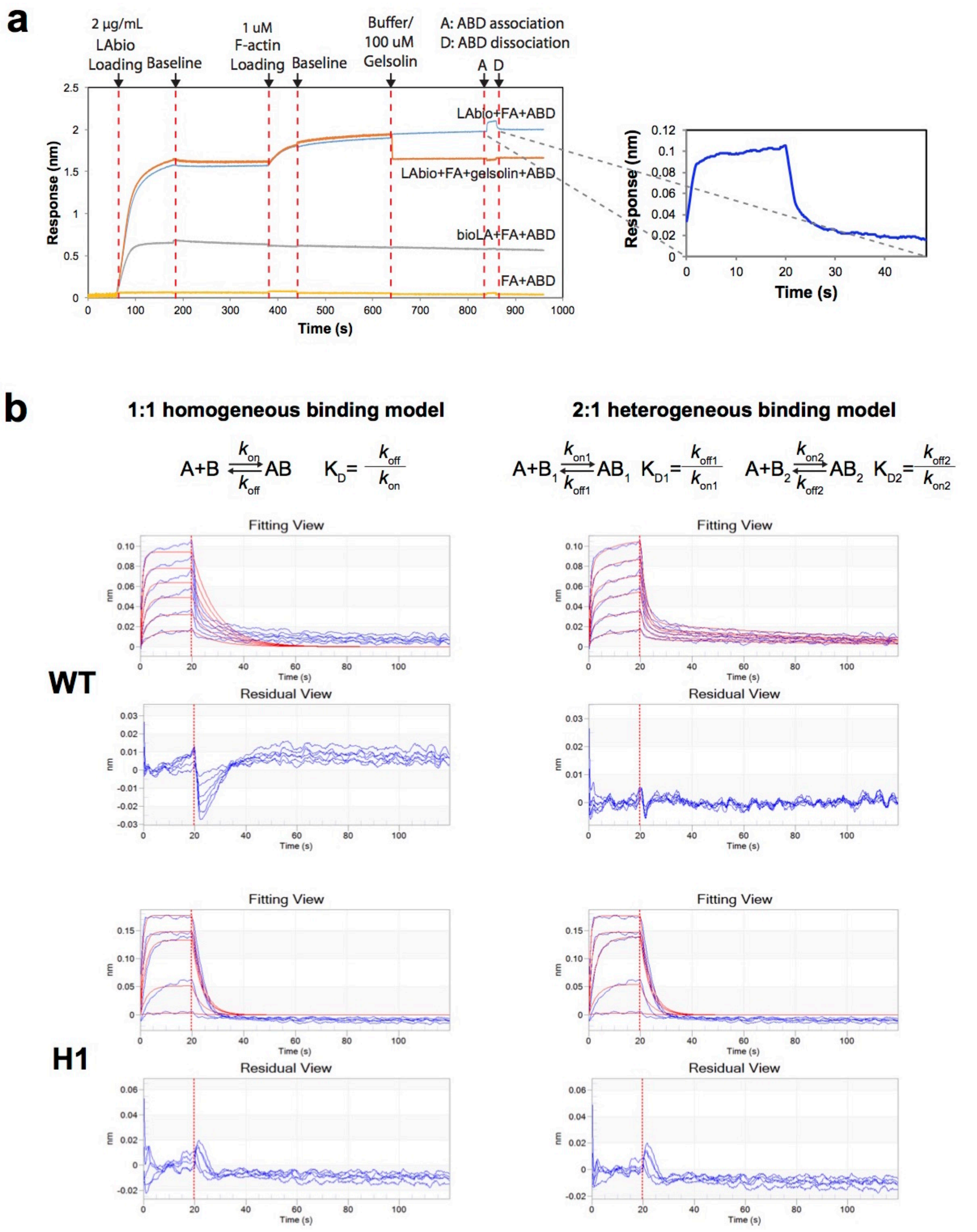

Supplementary Figure 13: Bio-layer interferometry (BLI) analyses of the αEcat-ABD-F-actin interaction.

a. Comparison of BLI response curves. Biosensors coated with LAbio (the C-terminally biotinylated LifeAct; blue and red), bioLA (the N-terminally biotinylated LifeAct; gray) or

buffer (yellow) was used to immobilize F-actin (FA), and measure subsequent association (A) and dissociation (D) of αEcat-ABD. The binding of F-actin to LAbio-coated sensors was confirmed by increased response curve upon stable association with F-actin. Incubation of a SA-sensor-LAbio-FA with gelsolin S1 resulted in depolymerisation of bound F-actin and the response curve returning to the level before the addition of F-actin, and did not show any interaction with ABD.

b. Comparison of the fit of 1:1 homogeneous and 2:1 heterogeneous ligand (HL) binding models with the αEcat-ABD-WT and -H1 BLI data. Both the fitting and residual (a plot of the difference between the response and fitted curves) views of the WT data suggest that a 2:1 HL binding model is suited to fit the WT data than the 1:1 homogenous binding model (Residual view shows greater deviations between the response and fitted curves). In comparison, the shape of H1 response curves are noticeably different from the WT curves, and the H1 data fit well with the 1:1 binding model. The 2:1 HL binding model fits similarly well with the H1 data, but the calculated percentage of two kinetic interactions in the total binding revealed that the best fit was obtained when there was no contribution from the second set of kinetic interaction, hence confirming the good agreement between the H1 data and the 1:1 fitting model.

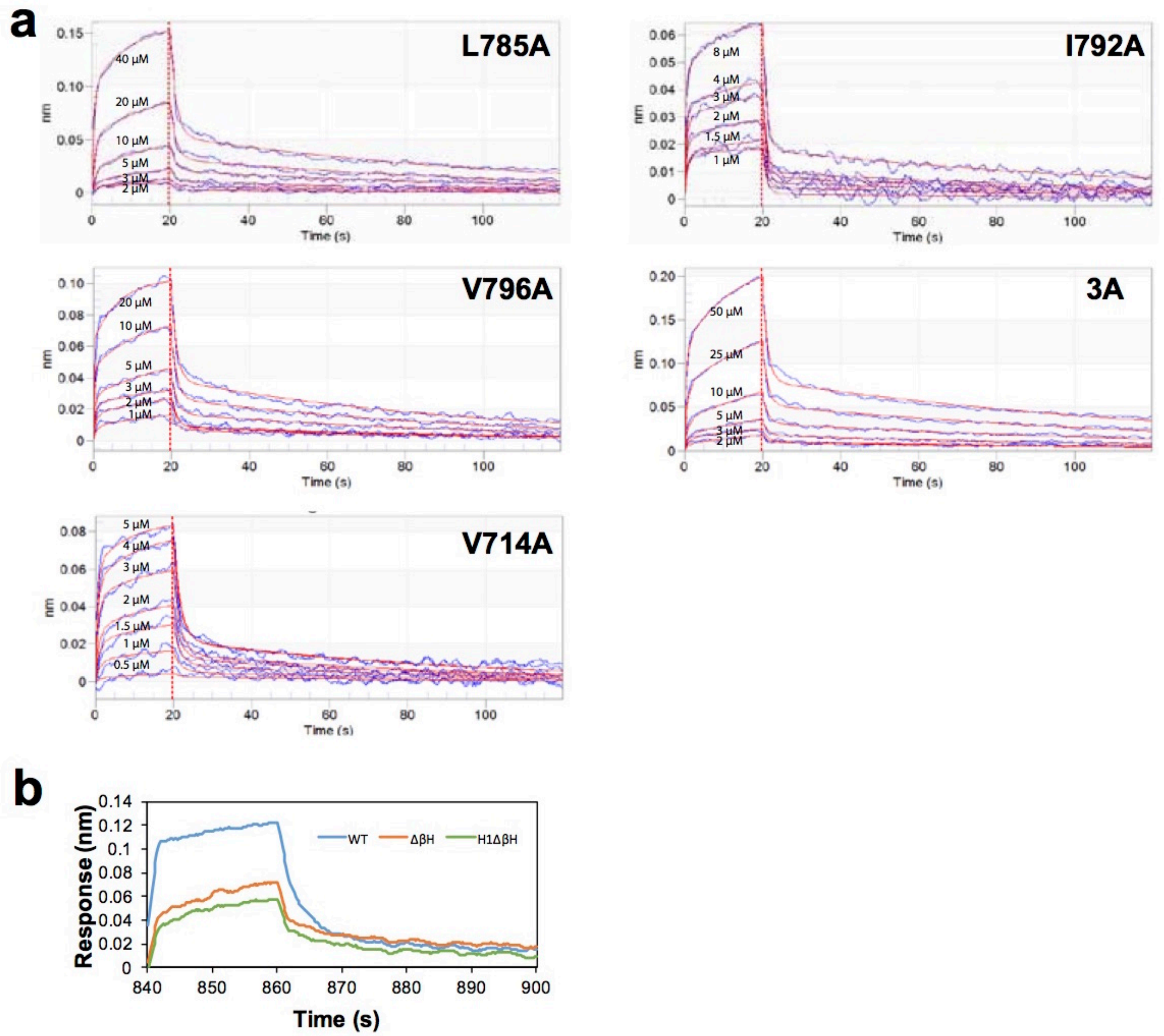

Supplementary Figure 14: BLI analyses of the interaction between F-actin and αEcat-ABD variants.

a. BLI responses curves of αEcat-ABD variants, L785A, I792A, V796A, 3A, and V714A. The K_D values were obtained by fitting concentration-dependent F-actin binding curves (blue) to a 2:1 heterogeneous binding model (red curves). Kinetic rate constants (k_{on} and k_{off}) and the dissociation constant (K_D) determined from curve fitting analyses are shown in Table 1.

b. BLI response curves of the αEcat-ABD variants containing the ΔβH mutation. Comparison of response curves obtained with 4 µM αEcat-ABD-ΔβH and -H1ΔβH mutants confirmed that the βH deletion affected the F-actin binding affinity, as suggested by actin cosedimentation results (Fig. 2e).

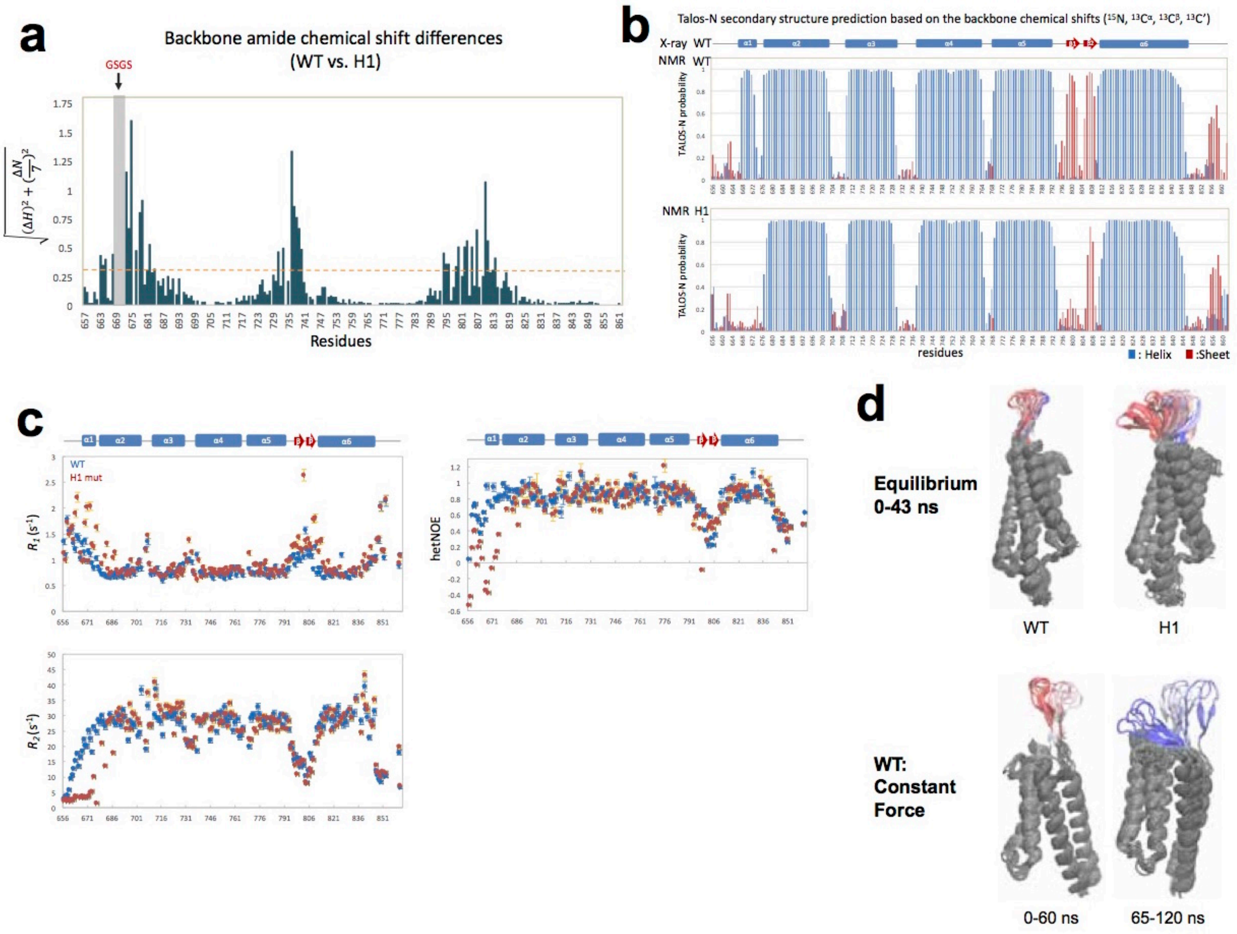

Supplementary Figure 15: NMR studies of the αEcat-ABD-WT and -H1 variants.

a. Plots of backbone amide chemical shift differences between αNcat-ABD-WT and αNcat-ABD-H1. The H1 mutation affected three regions: α1-helix, residues 732_N-741_N that interact with the side chain of $R670_N$ from α1-helix, and residues 795_N-815_N containing $V796_N$ and βH.

b. Chemical shift index-based secondary structure predictions of αNcat-ABD-WT and αNcat-ABD-H1. These predictions suggest that the region containing $V796_N$ in αNcat-ABD-H1 adopted an alternate conformation compared to αNcat-ABD-WT.

c. NMR relaxation studies of αNcat-ABD-WT and αNcat-ABD-H1. The βH motif displays fast tumbling motion in solution along with N- and C-termini.

d. Time-lapse images of the αNcat-ABD during MD simulations. Each β-hairpin (βH) domain is color-coded according to simulation timestep. As the simulation proceeds, the color changes from red to white to blue. The remaining ABD is colored gray. Residues N-terminal to α2-helix are omitted for clarity. (TOP) αNcat-ABD-WT and αNcat-ABD-H1 during equilibrium MD simulations. The time-lapse images show each frame (every 1 ns) during the 43-ns simulation of H1 or the first 43 ns of the simulation of αNcat-ABD-WT. (BOTTOM) αNcat-ABD-WT before (0-60 ns) or after (65-120 ns) α1-helix unfolds in a constant-force SMD simulation. The time-lapse images show every 5 frames (every 5 ns) of the SMD simulation of αNcat-ABD-WT.

Supplementary Table 1: α-catenin constructs

α-catenin	Constructs	Amino Acids Present	Missense Mutation
**Mouse αE-catenin**			
	αEcatFL	1-906	-
	αEcat-H1	1-906	RAIM670-673GSGS
	αEcat-ΔβH	1-798/811-906	-
	αEcat-H1ΔβH	1-798/811-906	RAIM670-673GSGS
	αEcat-ΔABD	1-651	-
	αEcat-ABD-WT	652-906	-
	αEcat-ABD-H1	652-906	RAIM670-673GSGS
	αEcat-ABD-Δα1	671-906	-
	αEcat-ABD-ΔβH	652-798/811-906	-
	αEcat-ABD-H1ΔβH	652-798/811-906	RAIM670-673GSGS
	αEcat-ABD-V714A	652-906	V714A
	αEcat-ABD-L785A	652-906	L785A
	αEcat-ABD-I792A	652-906	I792A
	αEcat-ABD-V796A	652-906	V796A
	αEcat-ABD-3A	652-906	L785A/I792A/V796A
	αEcat-ABD-H1L785A	652-906	RAIM670-673GSGS+L785A
	αEcat-ABD-H1I792A	652-906	RAIM670-673GSGS+I792A
	αEcat-ABD-H1V796A	652-906	RAIM670-673GSGS+V796A
	NM_IABD	1-402/663-906	-
	NM_IABD*	1-402/697-906	-
	NM_I	1-402	-
	ABD	663-906	
	ABD*	697-906	
**Mouse αN-catenin**			
	αNcat-ABD-WT	651-905	-
	αNcat-ABD-H1	651-905	RAIM669-672GSGS
	αNcat-ABD-Δα1	670-905	-
	αEcat-ABD-L785A	651-905	L784A
	αEcat-ABD-I792A	651-905	I791A
	αEcat-ABD-V796A	651-905	V795A
	αEcat-ABD-3A	651-905	L784A/I791A/V795A
**Drosophila α-catenin**			
	αCatFL	1-917	-
	αCat-H1	1-917	REAM683-686GSGS
	αCat-Δα1	1-658/691-917	-
	αCat-ΔβH	1-811/824-917	-
	αCat-H1ΔβH	1-811/824-917	REAM683-686GSGS
	αCat-3A	1-917	L798A/I805A/V809A
	αCat-ΔABD	1-708	-

Supplementary Table 2: Crystallographic data statistics

	αNcat-ABD-H1 Form A	αNcat-ABD-H1 Form B	αEcat-ABD-WT Native	αEcat-ABD-WT Br-SAD
**Data Collection**				
Space group	$P\ 6_522$	$P\ 2_12_12_1$	$P\ 2_12_12_1$	$P\ 2_12_12_1$
Cell dimensions				
a, b, c (Å)	108.7, 108.7, 133.1	75.1, 82.8, 105.3	56.3, 80.6, 104.5	56.3, 80.6, 104.5
α, β, γ (°)	90, 90, 120	90, 90, 90	90, 90, 90	90, 90, 90
Wavelength (Å)	1.0	1.0	1.0	0.9198
Resolution range (Å)	42-2.2 (2.28-2.20)[a]	43-2.8 (2.91-2.8)	46-2.2 (2.28-2.20)	50-2.3 (2.34-2.30)
R_{merge}(I) (%)	7.6 (68.0)	7.2 (68.7)	5.9 (52.2)	6.9 (47.8)
I /σ(I)	31.7 (3.6)	21.7 (2.8)	31.7 (3.8)	29.0 (3.9)
$CC_{1/2}$	0.98 (0.89)	0.97 (0.88)	0.99 (0.93)	0.98 (0.93)
Number of reflections	24191	16455	24938	21694
Completeness (%)	99.8 (100.0)	99.9 (100.0)	100.0 (100.0)	100.0 (100.0)
Redundancy	13.1 (13.3)	7.3 (7.4)	7.4 (7.4)	7.3 (7.4)
FOM				0.21
No. of Br sites				8
**Refinement**				
R_{cryst}/R_{free} (%)	20.2/22.6	19.9/25.5	20.6/25.2	
No. atoms				
Protein	1472	2897	2955	
Ion	-	-	15	
Water	89	19	97	
B-factors (Å^2)				
Protein	54.4	43.8	51.3	
Ion	-	-	73.3	
Water	57.9	35.9	49.5	
Ramachandran statistics				
Most favorable regions (%)	99.5	95.8	98.2	
Allowed regions (%)	0.0	3.4	1.9	
Disallowed regions (%)	0.5	0.8	0.0	
R.m.s deviations				
Bond lengths (Å)	0.008	0.009	0.008	
Bond angles (∘)	0.79	0.92	0.84	

[a] Values in parentheses are for the highest resolution shell.
