# Supplementary material for: Force-dependent allostery of the α-catenin actin-binding domain controls adherens junction dynamics and functions
Source: Nat Commun. 2018 Nov 30;9:5121. doi: 10.1038/s41467-018-07481-7 (PMC6269467; doi:10.1038/s41467-018-07481-7)
Supplement: Supplementary file 3 — Description of Additional Supplementary Files [file 41467_2018_7481_MOESM3_ESM.pdf]

Description of Additional Supplementary Files

File Name: Supplementary Movie 1
Description: A movie of constant-force SMD simulations of αNcat-ABD-WT. Cartoon representation shows that the α1-helix (blue) starts to unfold at ~60 ns

File Name: Supplementary Movie 2
Description: A close-up view of constant-force SMD simulations of αNcat-ABD-WT. $V796_N$ flips out from the interior pocket (red and blue surface) shortly before the α1-helix (blue surface and ribbon) unfolds and pulls away from the pocket at ~60 ns.

File Name: Supplementary Movie 3
Description: Wide-field fluorescence spinning-disk live-cell microscopy of αEcatFL-mGFP (WT) or αEcat-H1-mGFP (H1) expressing R2/7 cells. Cells stably expressing αcat-mGFP lines were infected with LifeAct Ruby, resulting in some cells not displaying the Ruby signal. Cells were plated on glass-bottomed dish coverslips ∼48hr before imaging, and scratch-wounds were generated 18 hours before imaging. Time-lapse sequences were acquired at 30 s intervals for both GFP and Life-Act-Ruby channels. Cell line, time-stamp and scale bars (21 µm) are burned-into the movie sequence. A six-panel composite of grayscale inverted and color overlay movies are shown.

File Name: Supplementary Movie 4
Description: Phase contrast live imaging of scratch wounded R2/7 cell monolayers. A four-panel composite of image sequences is shown for αEcatFL (WT), αEcat-H1 (H1), αEcatFL-ΔβH (WTΔβ) and αEcat-H1ΔβH (H1Δβ)-expressing cells. Time-lapse sequences were acquired at 10-min intervals for 15 hours.
